# Representation Learning for Class C G Protein-Coupled Receptors Classification

**DOI:** 10.3390/molecules23030690

**Published:** 2018-03-19

**Authors:** Raúl Cruz-Barbosa, Erik-German Ramos-Pérez, Jesús Giraldo

**Affiliations:** 1 Computer Science Institute, Technological University of the Mixteca Region, 69000 Huajuapan, Oaxaca, Mexico; erik@mixteco.utm.mx; 2 Laboratory of Molecular Neuropharmacology and Bioinformatics, Institut de Neurociències and Unitat de Bioestadística, Universitat Autònoma de Barcelona, 08193 Bellaterra, Spain; 3 Network Biomedical Research Center on Mental Health (CIBERSAM), Universitat Autònoma de Barcelona, 08193 Bellaterra, Spain

**Keywords:** representation learning, G protein-coupled receptors, deep learning, pattern classification

## Abstract

G protein-coupled receptors (GPCRs) are integral cell membrane proteins of relevance for pharmacology. The complete tertiary structure including both extracellular and transmembrane domains has not been determined for any member of class C GPCRs. An alternative way to work on GPCR structural models is the investigation of their functionality through the analysis of their primary structure. For this, sequence representation is a key factor for the GPCRs’ classification context, where usually, feature engineering is carried out. In this paper, we propose the use of representation learning to acquire the features that best represent the class C GPCR sequences and at the same time to obtain a model for classification automatically. Deep learning methods in conjunction with amino acid physicochemical property indices are then used for this purpose. Experimental results assessed by the classification accuracy, Matthews’ correlation coefficient and the balanced error rate show that using a hydrophobicity index and a restricted Boltzmann machine (RBM) can achieve performance results (accuracy of 92.9%) similar to those reported in the literature. As a second proposal, we combine two or more physicochemical property indices instead of only one as the input for a deep architecture in order to add information from the sequences. Experimental results show that using three hydrophobicity-related index combinations helps to improve the classification performance (accuracy of 94.1%) of an RBM better than those reported in the literature for class C GPCRs without using feature selection methods.

## 1. Introduction

G protein coupled receptors (GPCRs) are integral cell membrane proteins responsible for translating the molecular signals encoded in the chemical structure of hormones and neurotransmitters from outside to inside the cell. GPCRs share a common structure consisting of seven transmembrane helices (7TM), which are linked by three extracellular and three intracellular loops [1]. The binding of endogenous or synthetic agonists causes the activation of the receptor, which results in conformational changes that allow the allosteric coupling of accessory proteins such as G protein or β-arrestin at the intracellular part of the receptor [2,3]. Activation of these accessory proteins triggers the series of steps that constitute the signal transduction mechanism, which eventually lead to the observed physiological responses. The human GPCRs have been classified into five main families or classes (glutamate or class C, rhodopsin or class A, adhesion, frizzled or class F/taste2 and secretin or class B) by phylogenetic analysis [4]. Crystallographic determinations of a number of ligand-GPCR complexes have provided insights into the recognition determinants that discriminate between agonists (activators) and antagonists (inhibitors) [5], whereas other techniques such as nuclear magnetic resonance (NMR) [6], fluorescence approaches [7] and molecular dynamics (MD) [8] have led to mechanistic proposals for receptor activation and the allosteric transmission of the signal from the ligand binding site to the G protein or β-arrestin binding sites of the receptor.

GPCRs are at the center of current drug discovery programs. As of November 2017, approximately 35% of approved drugs in the United States or European Union target GPCRs [9]. There are different criteria for therapeutic drug design. One is selectivity, as it seems appropriate that drugs act selectively through specific receptors. Another is the concept of receptor polypharmacology in which a drug exerts a combination of positive effects by binding to different receptors [10]. Notwithstanding the approach that is followed, the correct classification of receptors in public databases is fundamental for virtual screening studies and in the examination of receptor functionality in general. To this aim, machine learning methods have proven to be useful [11,12,13,14,15,16,17]. For this, the standard procedure follows a feature extraction stage, where many ad hoc representations designed by specific domain experts can be used, and then, a classification stage is utilized. For the first stage, there are two main approaches to analyze GPCR sequences in order to extract the inherent features of the original sequences: multiple alignment and alignment-free representations. Many methods of both techniques have been developed in the literature achieving good representations, which are confirmed by the corresponding classification results [11,12,13,14,18,19,20]. However, the obtained/extracted representations are domain-dependent, which considers only certain factors (as frequency, order, etc.) of the original sequences.

In recent years, the representation learning field has arisen as an alternative resource for learning representations of the data that makes it easier to extract useful information when building classifiers [21]. That is, the main idea is to extract the relevant features (explanatory factors) from the observed data without using feature engineering methods. Following this idea and the good results presented in [22,23,24,25,26], in this paper, we aim to use a deep architecture in order to implicitly represent the explanatory factors of the protein sequences as much as possible and at the same time to obtain a model for classification. To this aim, we propose to use aligned GPCR sequences, which are translated into a numeric form by using an amino acid property index [27]. In the first stage, a hydrophobicity-related index is selected (because of its importance in determining the structure and function of GPCRs [14]) as the input for several deep architectures in order to choose one of them and find its parameters. After that, the preprocessed amino acid index (AAindex) database [19] is used as the input for training the selected deep architecture in order to implicitly represent the explanatory factors of the protein sequences. Experimental results assessed by the classification accuracy, Matthews’ correlation coefficient (MCC) and balanced error rate (BER) show that using the hydrophobicity index number 531 and a restricted Boltzmann machine (RBM) can achieve performance results (accuracy of 92.9%) similar to those reported in the literature [12,20].

As a second proposal, we hypothesize that using two or more physicochemical property index combinations instead of only one might add information from the sequences that a deep architecture can extract and classify in a better way. Experimental results show that using three hydrophobicity-related index combinations helps to improve the classification performance (accuracy of 94.1%) of an RBM better than those reported in the literature for class C GPCRs without using feature selection methods. The class C subfamily has been chosen for the present study due to structural, functional and therapeutic reasons [28].

## 2. Materials and Methods

### 2.1. Datasets

The current study focuses on class C GPCRs, which have become an increasingly important target for new therapies, particularly in areas such as fragile-X syndrome, schizophrenia, Alzheimer’s disease, Parkinson’s disease, epilepsy, L-DOPA-induced dyskinesias, generalized anxiety disorder, migraine, chronic pain, gastroesophageal reflux disorder, hyperparathyroidism, osteoporosis and drug addiction [29].

Because of its specificity, data were taken from GPCRdb (http://gpcrdb.org/) [30], which is defined as a molecular-class information system that collects, combines, validates and disseminates large amounts of heterogeneous data on GPCRs [31]. GPCRdb divides the GPCR superfamily into 5 families: the class A Rhodopsin like, the class B secretin like, the class C metabotropic glutamate/pheromone, vomeronasal receptors (V1R and V3R) and taste receptors (T2R).

Class C GPCRs were selected for analysis because of (i) their structural complexity, (ii) high sequence length variability and (iii) therapeutic relevance. Briefly, (i) whereas all GPCRs are characterized by sharing a common seven-transmembrane (7TM) domain, responsible for G protein/β-arrestin activation, most class C GPCRs include, in addition, an extracellular large domain, the Venus flytrap (VFT) and a cysteine rich domain (CRD) connecting both [28]. It was till 2014 that the crystal structures of the 7TM domains of two class C receptors had been solved [32,33]. (ii) The full or partial presence of the whole domain structure confers a high sequence length variability to this family. (iii) The involvement of class C GPCRs in many neurological disorders, as previously mentioned, makes this class an attractive target for drug discovery and development.

Class C is, in turn, subdivided into seven types: metabotropic glutamate (mG), calcium sensing (Cs), $GABA_{B}$(gB), vomeronasal (Vn), pheromone (Ph), odorant (Od) and taste (Ta). The investigated dataset is available in two forms: unaligned and aligned versions, which can be downloaded as Supplementary Material files. The former and the latter are distributed as shown in Table 1 and Table 2, respectively. The unaligned version is used for experimentation with alignment-free transformations, while the aligned one is used for experimentation with representation learning methods.

When the aligned version is used, each sequence is converted to a basic and numeric form by using an amino acid physicochemical property index taken from the amino acid index (AAindex) database [27]. This database contains three sections: AAindex1, AAindex2 and AAindex3 (Version 9), where AAindex1 contains 544 indices. For our experimentation, we used a preprocessed version of AAindex1, which contains 531 indices. All of them are available as Supporting Information in [19].

### 2.2. GPCR Representations

There are two main approaches to analyzing GPCR sequences through machine learning methods in order to capture the inherent features of the original sequences: (a) multiple alignment and (b) alignment-free representations. Both of them have been extensively utilized depending on the final application or use. Many methods of both techniques have been developed in the literature achieving good representations, which are confirmed by the corresponding classification results [11,12,13,14,18,19,20]. However, most of them are manually designed ad hoc by specific domain experts as a pre-processing step, which produces the fixed-length inputs for the classification methods. Therefore, the obtained/extracted representations are domain-dependent, which considers only certain factors (such as frequency, order, etc.) of the original sequence. Consequently, the extracted features can be relevant or not when they are used for different applications.

#### Multiple Sequence Alignment and Alignment-Free Representations

A very common preprocessing step for protein classification is multiple sequence alignment (MSA). The outputs of MSA are sequences of the same length using the one-letter code of the amino acids. Several methods and tools of MSA have been developed for studies of homology and evolutionary relationships between the sequences [34,35,36]. In addition, MSA output can be used as input for machine learning methods applied to classification tasks. Usually, the MSA output is directly used with natural language processing (as n-grams) or similarity matrix-related techniques. When MSA is used, the protein classification results strongly depend on the characteristics of the information provided by the alignment.

On the other hand, alignment-free protein representations have been defined in the literature in order to capture as much relevant information that might be conveyed by an amino acid sequence as possible. Among these, some rely on transformations based on the amino acid physicochemical characteristics, such as the auto-cross-covariance transformation [37,38].

In this paper, we consider a basic and three advanced alignment-free data transformations to obtain fixed-length vectors as input data for supervised classification algorithms. The corresponding transformed resulting datasets are available as Supplementary Material files. The first and most simple one reflects the amino acid composition (AAcomp) of the primary sequence: the relative frequencies of the occurrence of the 20 amino acids are calculated for each sequence resulting in a N×20 matrix, where *N* is the number of sequences in the dataset. This transformation does not take into account the relative position of amino acids in the sequence.

The second and third are extensions of the AAcomp, which include sequence-order information. The second is known as pseudo-amino acid composition (PseAA) [39], while the third is formed by a hybrid feature vector, which combines multiscale energy (MSE) and PseAA representations. Both representations have shown a better GPCR classification performance than AAcomp [14,16].

For a GPCR sequence $S=R_{1},R_{2},\ldots ,R_{L}$ where $R_{i}$ represents the amino acid at position *i* in the sequence *S* of length *L*, the PseAA is defined as:

(1) $$\mathrm{PseAA}=[P_{1},P_{2},\ldots ,P_{20},\ldots ,P_{\Lambda }],$$

where Λ=20+n× λ (λ=0,1,…,m is the number of levels used to compute the correlation factors of the amino acids in the sequence, and *n* is the number of physicochemical properties used as relevant information for the GPCR sequences). Following [14,40,41], we set λ=21 as the maximum level and n=2 physicochemical properties (hydrophobicity and hydrophilicity). That is, the PseAA feature vector length is 62, where the first 20 elements are the relative frequencies of occurrence of the 20 amino acids (as AAcomp), and the remaining elements are the first-level to λ-level correlation factors of amino acid sequences for each physicochemical property. In our case, the PseAA transformation of the class C GPCRs was obtained by using the PseAAC server [42].

Now, the wavelet-based MSE representation of a sequence is defined as:

(2) $$MSE\left(k\right)=\left[d_{1}^{k},d_{2}^{k},\ldots ,d_{m}^{k},a_{m}^{k}\right],$$

where k=1,2,…,N (*N* is the total number of GPCRs); $d_{i}^{k}$ is the root mean square energy of wavelet detail coefficients in the corresponding *i*-th scale; and $a_{m}^{k}$ is the root mean square energy of wavelet approximation coefficients in the *m*-th scale. For this transformation, the GPCR sequences are first converted into a numeric form by using hydrophobicity values taken from the FHscale [43]. The resulting numeric form takes the role of a digital signal in which the wavelet (Haar) transformation is applied. That is, the approximation ($a_{m}^{k}$) and detailed ($d_{i}^{k}$) coefficients are computed, where the maximum decomposition level (scale) *m* of a sequence is taken as $\log_{2}\left(L\right)$.

Finally, the MSE and PseAA are concatenated to form a hybrid feature vector as follows:

(3) $$\mathrm{PseAA}-\mathrm{MSE}=[P_{1},P_{2},\ldots ,P_{20},\ldots ,P_{\Lambda },d_{1}^{k},d_{2}^{k},\ldots ,d_{m}^{k},a_{m}^{k}].$$

Major details for computing PseAA and MSE can be found in [14,16,40].

The fourth representation, related by the descriptors obtained in [44], is the ACC transformation [37,38]. Here, time series models are applied to the protein sequences in order to extract their sequential patterns, and consequently, the extracted information is sequence-order dependent. This representation was originally developed in [38] and then applied and modified in [15,37].

The ACC transformation can be described as follows: each sequence is first translated into physicochemical descriptors by representing each amino acid with the five *z*-scales derived in [44], where these scales are in turn obtained from 26 physicochemical properties. The auto-covariance (AC) and cross-covariance (CC) variables are then computed from the transformed sequences. The AC measures the correlation of the same descriptor, *d*, between two residues separated by a lag, *l*, along the sequence, and it can be calculated as:

(4) $$AC_{d}\left(l\right)=\sum_{i=1}^{n-l}\frac{\left(v_{d,i}-{\bar{v}}_{d}\right)\left(v_{d,i+l}-{\bar{v}}_{d}\right)}{{\left(n-l\right)}^{p}}.$$

The CC variable measures the correlation of two different descriptors between two residues separated by a lag along the sequence, and it can be computed as:

(5) $$CC_{dd^{\prime }}\left(l\right)=\sum_{i=1}^{n-l}\frac{\left(v_{d,i}-{\bar{v}}_{d}\right)\left(v_{d^{\prime },i+l}-{\bar{v}}_{d^{\prime }}\right)}{{\left(n-l\right)}^{p}},$$

where l=1,…,Lag and Lag is the maximal lag, which must be lesser than the length of the shortest sequence in the dataset; *n* is the total number of amino acids in the sequence; $v_{d,i}$ is the value of descriptor d=1,…,D (D=5) of an amino acid in a sequence at position *i*; ${\bar{v}}_{d}$ is the mean value of descriptor *d* across all positions; and *p* is the degree of normalization.

From these, the ACC fixed-length vectors are obtained: first, the AC and CC terms from Equations (4) and (5) are concatenated for each lag (C(l)=[AC(l)CC(l)]), and then, the ACC is obtained for a maximum lag Lag by concatenating the C(l) terms, that is,

(6) ACC(Lag)=[C(1),…,C(Lag)].

Here, the length of an ACC feature vector is length(AC)×length(CC)×Lag=25×Lag. Details of this procedure can be found in [15,37].

### 2.3. GPCR Feature Learning Proposal through the Deep Approach

In recent years, the representation learning field has arisen as an alternative resource for learning representations of the data that makes it easier to extract useful information when building classifiers [21]. That is, the main idea is to extract the relevant features (explanatory factors) from the observed data without using feature engineering methods.

When representation learning methods are applied to GPCR sequences, a fixed-length and as unprocessed as possible representation of them is needed as the input for these methods. For this reason, we take the aligned version (see Table 2) with 259 fixed-length sequences of the GPCR database described in [30].

In our first proposal, each aligned sequence is converted to a basic and numeric form by using an amino acid property index taken from the preprocessed AAindex1 database [19,27]. That is, the sequence $S=R_{1},R_{2},\ldots ,R_{L}$ of length *L* is now represented by:

(7) $$S^{\prime }=I_{1}^{k},I_{2}^{k},\ldots ,I_{L}^{k}$$

where $I_{i}^{k}$ indicates the corresponding numeric value of the amino acid $R_{i}$ using the *k*-th amino acid property index. In the case that a gap is presented in a sequence, it is replaced by a zero value. From Equation 7, it is observed that neither occurrence frequency, nor order information from *S* are included in $S^{\prime }$.

**Figure 1:**
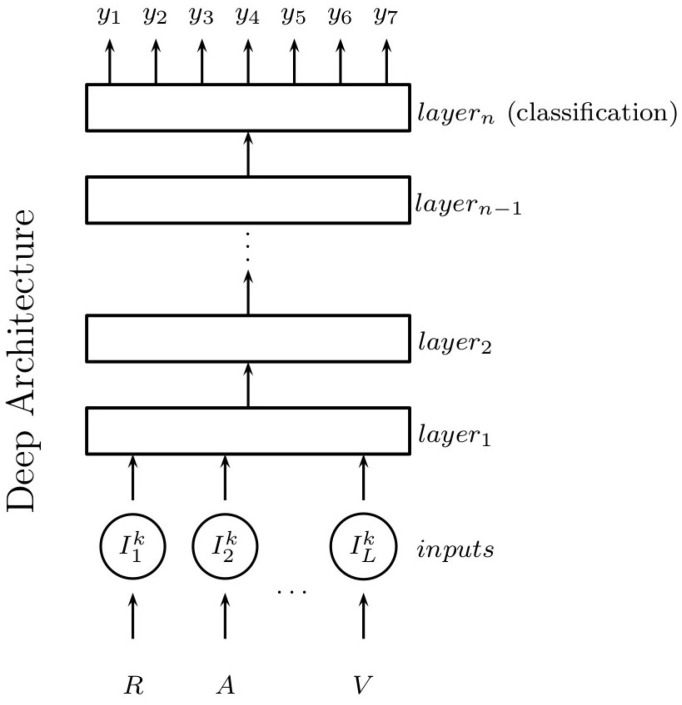
Deep architecture training proposal scheme.

In this way, for the class C GPCRs dataset, we form k=1,2,…K input datasets, where K=531 is the total number of indices of the preprocessed [19] amino acid properties index database [27]. Each *k*-th dataset is used as input for training a deep architecture in order to implicitly represent the explanatory factors of the protein sequences as much as possible and at the same time to obtain a model for classification. For illustration, Figure 1 shows how a sequence is used for training a deep architecture. It is observed from this figure that we can use different kinds of deep architectures to represent a dataset. In this paper, we experiment with basic and functional architectures, namely: (a) autoencoders, (b) convolutional neural networks (CNN) and (c) restricted Boltzmann machine in the first stage in order to select the architecture that best represents the original dataset. In this stage, a hydrophobicity-related index is selected because of its importance in determining the structure and function of GPCRs [14].

After the selection of a deep model, we proceed to find the right number of hidden layers and the number of neurons in each hidden layer by using a grid search in the range of [1,2,…,10] and [100,200,300,500,800], respectively. The number of neurons in a hidden layer is selected to be lesser or greater than the number of input neurons in order to allow the codification or magnification of the information from the inputs.

Once the number of hidden layers and the number of neurons in each hidden layer is found, we look into a neighborhood of the number of neurons in a hidden layer in order to refine and confirm the results. Next, we use the best setting (deep model, number of hidden layers and number of neurons in a hidden layer) to train a model using each one of the 531 indices from the AAindex database. This process will help to select the physicochemical index that in conjunction with the selected deep architecture represents the explanatory factors of the GPCR sequences.

Now, we hypothesize (as a second proposal) that using two or more physicochemical property indices instead of only one might add information from the sequences that a deep architecture can extract and classify in a better way. This is carried out by combining the physicochemical indices for each amino acid in a sequence. That is, if a GPCR sequence has a length of *L* (259), after combination, its length is L×n, where *n* is the number of indices. For n=2, the sequence is represented by:

(8) $$S^{\prime \prime }=I_{1}^{j},I_{1}^{k},I_{2}^{j},I_{2}^{k},\ldots ,I_{L}^{j},I_{L}^{k}$$

where $I_{i}^{j},I_{i}^{k}$ indicates the combination of the corresponding numeric value of the amino acid $R_{i}$ using the *j*-th and *k*-th amino acid property indices.

### 2.4. Deep and Conventional Supervised Learning Methods

In this paper, we experiment with basic and functional deep architectures, namely: autoencoders, convolutional neural networks and restricted Boltzmann machine in the first stage in order to select the architecture that best represents the original dataset. These architectures help to discover complex structures in datasets, which are used to compute the representations in each layer. These distributed representations lead to improved generalization for different tasks.

Convolutional neural networks have been widely applied to the recognition of objects in digital images. The architecture of a typical deep CNN is structured as a series of convolutional layers and pooling (subsampling) layers. The role of a convolutional layer is to detect local conjunctions of features from the previous layer, whereas the role of a pooling layer is to merge semantically similar features into one [45].

A stacked autoencoder is used mainly to encode the inputs into some representation so that the inputs can be reconstructed from that representation. In practice, the output representation can also be used to initialize a deep neural network for multi-class classification. In this paper, a stochastic version of the autoencoder is used, namely the denoising autoencoder, which avoids learning the identity function [46,47].

A stacked RBM is a particular type of energy-based model with hidden variables, which has the restriction that its neurons must form a bipartite graph. An RBM is formed by a visible input layer and a hidden layer and connections between them, but not within a layer. Usually, the contrastive divergence algorithm is utilized as the unsupervised training procedure to detect features from the inputs [46,48].

For classification tasks, a deep belief network or simply a deep neural network can be constructed by stacking RBMs or autoencoders where the top layer (*n*) is used as the classifier’s output. In the first stage, a deep network of this kind is trained without supervision using n−1 layers to detect the main features of the inputs. After this, the *n*-th layer is added to the network and trained with supervision through the error backpropagation algorithm to perform classification.

On the other hand, we also compare the obtained RBM results with some conventional classifiers such as *k*-nearest neighbor (*k*-NN), decision tree (DT), multilayer perceptron (MLP) and support vector machine (SVM).

*k*-NN is one of the simplest classifiers. It finds the *k* points in the training set that are nearest to the test input, then counts how many members of each class are present in the corresponding neighborhood (formed by the *k* points) and returns a class label belonging to the most common class in such a neighborhood.

Another basic classifier is a DT or classification tree. It partitions the feature space into hyperrectangles with sides parallel to the axes and then fits a simple model in each one. That is, the sequence of decisions is applied to individual features. In the resulting (tree-like) structure, an internal node represents a test on a variable or attribute, and a leaf node represents a class label.

MLP is a sophisticated feedforward neural network architecture, which can be trained in a supervised manner through the error backpropagation algorithm. The network contains layers of hidden neurons, which extract meaningful features from the input vectors. Each neuron in the network uses a nonlinear activation function, which helps to model non-linearities in its input-output relation.

A more sophisticated and widely-applied nonlinear classifier is SVM. It separates the input data points by mapping them into a high-dimensional feature space where a hyper-plane is constructed. This hyper-plane creates a decision surface, which has a maximum distance to the nearest points in the feature space. That is, two key concepts are involved in the design of an SVM: large margin separation and kernel functions. The former concept means that the constructed hyper-plane should be placed as far as possible away from the points in different classes. The latter concept helps to calculate the similarity between points in the corresponding feature space, which allows an SVM to generate nonlinear decision boundaries [49,50]. In this paper, a radial basis function was used as the kernel (due to the good results presented in [12,20]), where a grid search was carried out to find the regularization parameter *C* and the kernel width parameter σ.

#### Performance Assessment Measures

The performance measures used in the experiments are classification accuracy, MCC and BER. Accuracy is widely known and used as the proportion of correctly-classified cases. MCC and BER are commonly used as performance measures when the analyzed datasets are class-unbalanced. All of them can be naturally extended from the binary to the multi-class context [51].

Let us assume a classification problem with S samples and *G* classes and two functions defined as tc,pc:S→{1,…,G}, where tc(s) and pc(s) return the true and the predicted class of *s*, respectively. The corresponding square confusion matrix *C* is:

(9) $$C_{ij}=\left|\left\{s\in S:tc\left(s\right)=i\;\mathrm{and}\;pc\left(s\right)=j\right\}\right|,$$

in which the ij-th entry of *C* is the number of cases of true class *i* that have been assigned to class *j* by the classifier. Then, the confusion matrix notation can be used to define the accuracy, MCC and BER as:

(10) $$\mathrm{accuracy}=\frac{\sum_{k=1}^{G}C_{kk}}{\sum_{i,j=1}^{G}C_{ij}},$$

(11) $$\mathrm{BER}=\frac{1}{G}\left(\Sigma_{i=1}^{G}\left[\frac{\Sigma_{j=1,j\neq i}^{G}C_{ij}}{\Sigma_{j=1}^{G}C_{ij}}\right]\right),$$

(12) $$\mathrm{MCC}=\frac{\sum_{k,l,m=1}^{G}C_{kk}C_{ml}-C_{lk}C_{km}}{\sqrt{\sum_{k=1}^{G}\left[\left(\sum_{l=1}^{G}C_{lk}\right)\left(\sum_{f,g=1f\neq k}^{G}C_{gf}\right)\right]}\sqrt{\sum_{k=1}^{G}\left[\left(\sum_{l=1}^{G}C_{kl}\right)\left(\sum_{f,g=1f\neq k}^{G}C_{fg}\right)\right]}}.$$

BER is the average of the errors on each class, which takes values in the interval [0,1]. Then, 0 means perfect classification where no error contribution per class was found, and 1 means an extreme misclassification case where items for each class are misclassified.

MCC is commonly used in the bioinformatics field and takes values in the interval [−1,1], where 1 means complete correlation (perfect classification), 0 means no correlation (all samples have been classified to be of only one class) and −1 indicates a negative correlation (extreme misclassification case). MCC is recommended as an optimal tool for practical tasks, since it presents a good trade-off among discriminatory ability, consistency and coherent behavior with a varying number of classes, unbalanced datasets and randomization [52].

## 3. Results and Discussion

The experimental results reported in this section aim to assess the ability of representation learning methods to extract the explanatory factors from the observed class C GPCR sequences without using feature engineering methods. For this purpose, two kinds of experimentation are designed.

Firstly, unaligned amino acid sequences are transformed according to the alignment-free transformations described in Section 2.2.1 in order to extract the relevant features that will help to gauge the classification performance using conventional supervised methods. Secondly, aligned amino acid sequences converted to a basic and numeric form are used as input for deep learning methods in order to implicitly represent the explanatory factors of the protein sequences. These models have the characteristic that at the same time the representation is extracted, a classification model is also obtained, which is assessed through classification performance.

### 3.1. Class C GPCRs Classification Using Alignment-Free Representations

The goal of the experiments in this subsection is two-fold. Firstly, we aimed to gauge the ability of the alignment-free amino acid sequence transformations to capture the inherent relevant features of class C GPCR subfamilies through supervised classification models. Secondly, we aimed to compare the performance of four conventional supervised models in terms of classification performance.

For the first set of experiments, the alignment-free transformations described in Section 2.2.1 are used in order to obtain the fixed-length feature vectors of the class C GPCRs unaligned dataset (see Table 1). This means that the AAcomp, PseAA, PseAA-MSE and ACC transformations are computed to obtain the corresponding four datasets as input for classification algorithms.

Following Section 2.2.1, a feature vector of the AAcomp dataset has a length of 20; for the PseAA dataset, the length is 62; and for the PseAA-MSE, the length is 74; taking a maximum decomposition level of m=11 ($\log_{2}\left(\max\left\{L_{1},L_{2},\ldots ,L_{N}\right\}\right)\approx 11$, where $L_{i}$ is the length of the sequence *i*). In the case of the ACC transformation, it uses two parameters that must be set to adequate values prior to classification: the maximum Lag and the degree of normalization *p*. In this study, we set both as Lag=13 and p=0.5, since the unaligned dataset is almost the same as in [11,12]. Then, the length of an ACC-transformed feature vector is 25×Lag=325.

For the second set of experiments, we selected two baseline and two sophisticated (non-linear) classifiers. Here, the *k*-nearest neighbor, decision tree, multilayer perceptron trained with the backpropagation algorithm and support vector machines were used. For *k*-NN, different neighborhoods were tried in the range k=1,…,10. Different settings for the number of hidden layers (hl) and number of neurons in a hidden layer (nhl) for MLP were used as hl=[1,2,3,4,5] and nhl=[10,20,30,40,50,60,70,80,90,100]. In the case of SVM classifier, the radial basis function kernel was used, which utilizes two parameters that must be identified in order to accurately predict unknown data: *C* and γ. For this, a grid search is carried out in the ranges C=[1,16] and $\gamma =[2^{-10},2^{5}]$ as in [12,20]. For these classifiers, only the parameters that lead to the best classification performance are reported.

For all conventional classifiers, the corresponding implementation available in the Weka (Version 3.6) toolbox [53] was used. It also allows data preprocessing where data normalization in the range [0,1] was carried out using the min−max normalization technique. In order to estimate the average classification performance, 10-fold cross-validation is used.

The average classification accuracy results using alignment-free representation datasets with the above described classifiers are shown in Table 3. From these results, SVM is shown to outperform the rest of the classifiers in terms of accuracy, which is similar to that reported in the literature [12,20].

On the other hand, the alignment-free transformation that best captures relevant features through classifiers is ACC, except for decision trees. This is followed by PseAA and PseAA-MSE transformation, which indicates the importance of adding sequence-order information in transformed feature vectors [11,14,16].

### 3.2. Class C GPCRs Classification Using Representation Learning

The goal of the experiments in this subsection is two-fold. Firstly, we aimed to gauge the ability of representation learning methods to extract the explanatory factors directly from the observed data sequences through deep learning approaches. Secondly, we aimed to compare the performance of deep and conventional learning models in terms of classification performance.

As stated in the first proposal of Section 2.3, the aligned dataset (see Table 2) is converted to a numeric form by using an amino acid property index taken from AAindex database [19,27]. From the 531 indices, in the first stage, we selected a hydrophobicity-related index, because of its importance in determining the structure and function of GPCRs [14], then the hydrophobicity index 2 is chosen. The resulting dataset is named AAhydro.

Three common deep architectures were selected for experimentation: autoencoders, restricted Boltzmann machines and convolutional neural networks. In order to select the best architecture, which will be tuned in a posterior step, a basic configuration for each was used: two hidden layers and 700 neurons for each layer. To estimate the classification performance of the deep models, stratified 10-fold cross-validation was carried out.

The corresponding implementation of deep architectures was taken from [54,55]. The average classification accuracy results of the different deep architectures are shown in Table 4. Here, it is observed that RBM outperforms the other deep architectures in terms of classification accuracy. It is worth noting that RBM is modeled through a Gaussian-Bernoulli distribution, which naturally allows real-valued inputs. Although it is widely-known that CNNs have good performance for image pattern recognition (where large datasets are used), this is not the case for class C GPCR classification where the amount of analyzed data is not large enough.

From here on, RBM is selected as the deep architecture trained with the backpropagation algorithm where gradient descent is accelerated by Nesterov’s method [56]. In order to find the right configuration for the number of hidden layers and the number of neurons for layer of RBM, an ad hoc and coarse grid search was carried out in the ranges [1,2,…,10] and [100,200,300,500,800], respectively. The corresponding average classification accuracy results of this search are progressively shown in Table 5, Table 6, Table 7 and Table 8.

From Table 5 and Table 6, it is observed that the right number of neurons for the first and second layer is around 500. Then, the number of neurons for the third and fourth layer is around 500. Table 7 and Table 8 show that no improvement is achieved when we add more hidden layers. We also tried five hidden layers, but the results are worse than previous tables; therefore, they are not reported.

Since the best results are obtained using 500 neurons for two hidden layers, we proceed with a fine grid search of around 500 neurons for each layer. Then, we tried the range [400, 450, 500, 550, 600] for each layer. The average classification accuracy results for this search are shown in Table 9. Again, the best results are obtained with 500 neurons for the first and second layers.

From previously-obtained results, we selected two hidden layers and 500 neurons for each layer as the right configuration for RBM. Now, we train the selected RBM architecture using each one of the 531 indices from the preprocessed AAindex database [19]. This process will help us to select the amino acid physicochemical property index that in conjunction with RBM represents the explanatory factors of the class C GPCR sequences.

The average classification results of the 12 amino acid physicochemical property indices with the highest classification accuracy are shown in Table 10. Since the resulting datasets are unbalanced (see Table 2), the MCC and BER measures are also presented in order to compare them with accuracy results.

It is observed from Table 10 that the amino acid property index number 531 in conjunction with RBM represents in a better way the explanatory factors of the class C GPCR sequences than the initial hydrophobicity index number 2. Although indices 531 and 485 have similar accuracy results, the BER measure is in favor of the hydrophobicity index number 531, which indicates a minimum mean misclassification for each class. Furthermore, this result is similar to that reported in the literature using feature engineering methods with SVM classifier (Konig 2013, 2014), but in contrast, an RBM learns representations directly from the observed data sequences.

**Figure 2:**
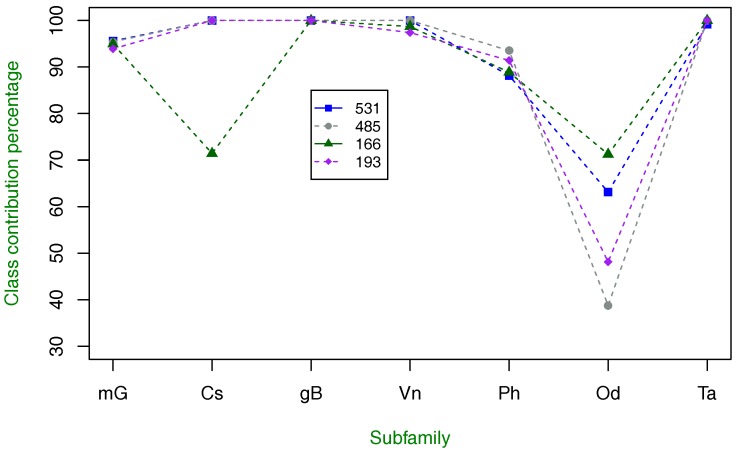
Class-specific percentage of contribution to overall classification using four amino acid property indices with the highest classification accuracy. metabotropic glutamate (mG), calcium sensing (Cs), $GABA_{B}$ (gB), vomeronasal (Vn), pheromone (Ph), odorant (Od) and taste (Ta).

In order to find out to what extent each of the seven class C GPCR types described in Section 2.1 can be discriminated from the rest and how each of them influences the overall classification performance, the four highest accuracy results represented by their corresponding amino acid property indices are presented in Figure 2 for all these types. Here, it is clear that the overall pattern of supervised classification is quite stable across amino acid property indices, except for index 166. The tendency is that the odorant and pheromone subfamilies are those that contribute less to the overall classification, which is a pattern similarly obtained in [11,12] with a difference (in favor of RBM) in the vomeronasal subfamily results. In this figure, five out of seven subfamilies (including vomeronasal) have high classification performance. The exception for this pattern is the results from index 166, which indicate that RBM cannot extract the explanatory factors of calcium sensing receptors, but in contrast, it has the highest recognition rate for the most difficult subfamily (odorant) to discriminate.

Results from Figure 2 suggest that if feeding an RBM with information of two or more amino acid property indices instead of one, it probably could extract and represent more inherent and hidden information from GPCR sequences and consequently improve classification performance. Therefore, as a second proposal, we can combine two or more amino acid property indices as inputs for the RBM architecture previously selected.

Providing two amino acid property indices to an RBM means that the input sequence is first converted to a numeric form as $I_{1}^{j},I_{1}^{k},I_{2}^{j},I_{2}^{k},\ldots ,I_{L}^{j},I_{L}^{k}$, where $I_{i}^{j},I_{i}^{k}$ indicates the combination of the corresponding numeric value of the amino acid *i* using the *j*-th and *k*-th amino acid property indices. For the next experiments, we combine pairs of indices from Table 10 in order to reduce the search space.

The average classification results of the five amino acid property index combinations with the highest classification accuracy are shown in Table 11. From this table, it is observed that a combination of indices 65 and 205 in conjunction with RBM can represent the class C GPCRs types in a better way than using only one index, then classification performance is improved and confirmed by all the performance measures. This result outperformed the one obtained in [12] using feature engineering methods and is similar to [20], obtained by feature selection methods, with the difference being that we did not resort to these kinds of methods.

**Figure 3:**
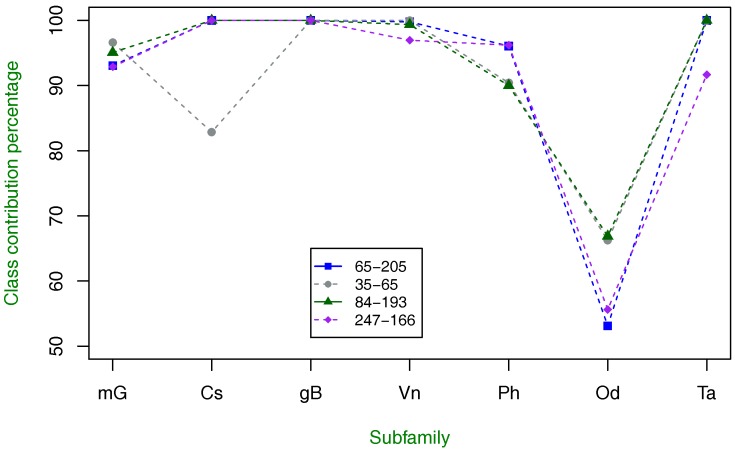
Class-specific percentage of contribution to overall classification using two amino acid property index combinations with the highest classification accuracy. The tendency of index combination results is similar to the results shown in Figure 2, but this time, the Ph and Od subfamilies are better discriminated.

As in the previous experiment, we investigate the class-specific contribution to overall classification for class C GPCR types, and this is shown in Figure 3. The tendency and pattern described by these results are very similar to the ones described by using only one amino acid property index, but this, time the recognition rate of the most difficult subfamily to discriminate is improved.

**Figure 4:**
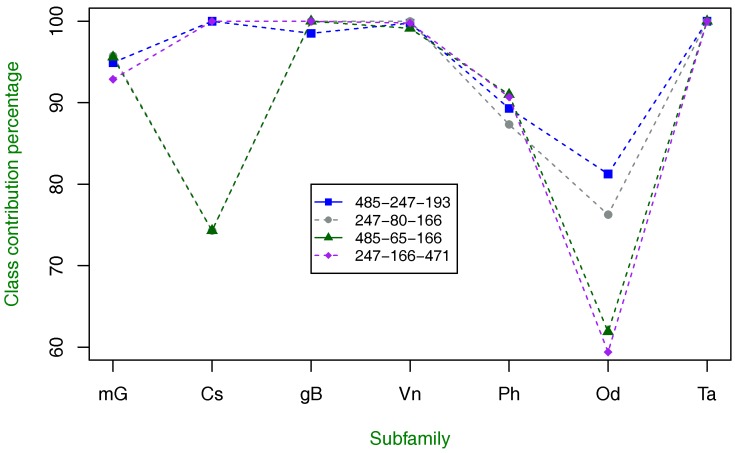
Class-specific percentage of contribution to overall classification using three amino acid property index combinations with the highest classification accuracy. As in Figure 2 and Figure 3, the tendency of index combination 485-247-193 is similar, but the recognition rate of the Od subfamily is highly improved.

Now, we proceed with combinations of three and more amino acid property index combinations. Since the results were not improved using four or more combinations, we only present the performance results with three index combinations in Table 12 and Figure 4. The classification results in Table 12 slightly improve the highest obtained using a combination of two indices. In particular, the combination of indices 485, 247 and 193 is better than the combination of 65 and 205 in terms of MCC and BER measures, but the rest of the combinations are not better than those shown in Table 11.

From Figure 4, it is observed that the same pattern described in Figure 2 and Figure 3 is found, including the recognition rate improvement of the odorant type.

**Figure 5:**
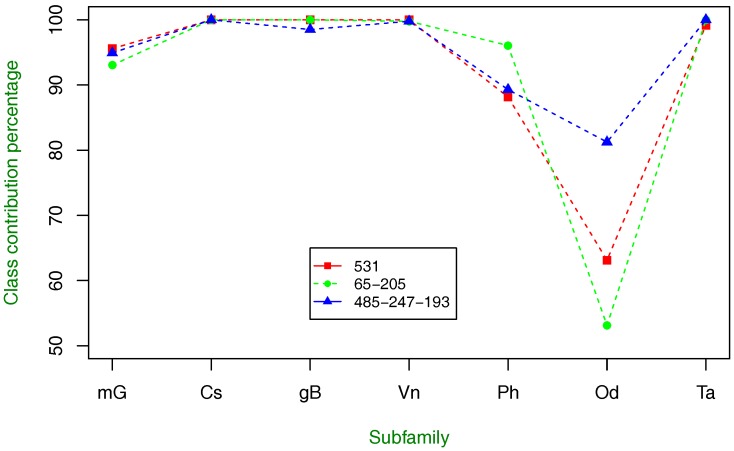
Class-specific percentage of contribution to overall classification using one, two and three amino acid property index combinations with the highest classification accuracy.

A summary of the highest classification performance using one, two and three amino acid property index combinations is presented in Figure 5. Here, it is observed that the highest results are addressed by the ability of the recognition (discrimination) of odorant and pheromone subfamilies. According to [11,12], subfamilies related to the odor function, such as vomeronasal, pheromone and odorant, are the most difficult to discriminate. However, Figure 5 shows that an RBM using one, two or three amino acid property index combinations can perfectly discriminate the vomeronasal type from the rest. Moreover, an RBM using the 485-247-193 index combination can also highly recognize the pheromone and odorant subfamilies. These results reveal the important contribution of hydrophobicity-related index combinations to correct amino acid sequence classification. This is not an unexpected result considering that GPCRs are membrane proteins, and thus, hydrophobic residues are highly present along the sequence and important both for receptor structure and function [14].

Finally, we compare the performance obtained with the highest classification accuracy results of RBM using one, two and three amino acid property index combinations with conventional supervised classification methods. For this purpose, the datasets obtained with one, two and three amino acid property index combinations are used as input for classification methods, such as SVM, *k*-NN and DT. The corresponding parameters of SVM and *k*-NN were as in Section 3.1, and the best average classification accuracy results are reported in Table 13.

From Table 13, it can be observed that RBM can extract and represent the inherent and hidden information of class C GPCRs in a better way than conventional classification methods, which is confirmed by the accuracy, MCC and BER measure results. These results outperformed those reported in the literature [11,12,20] for class C GPCR classification without using feature selection methods.

## 4. Conclusions

Given the interest in class C receptors in pharmacology and in the absence of much knowledge regarding their complete 3D crystal structures, the investigation of their functionality can be approached through the analysis of their primary structure in the form of amino acid sequences. For this, many works reported in the literature [11,13,14,16,19,20,37] have coincided with the fact that sequence representation is a key factor for the GPCR classification task. Following this idea and opposite to the standard procedure of applying feature engineering methods for sequence representation, the use of the representation learning approach for automatically acquiring the features that best represent the class C GPCR sequences is proposed in this paper. That is, the AAindex database is used as the input for training a stacked RBM in order to implicitly represent the explanatory factors of the protein sequences. Experimental results assessed by classification accuracy, MCC and BER show that using the hydrophobicity index number 531 in conjunction with an RBM can achieve performance results similar to those reported in the literature. Furthermore, it is also shown that using three hydrophobicity-related index combinations helps to improve the classification performance of an RBM better than those reported in the literature for class C GPCRs without using feature selection methods.

Besides, type-specific classification results have shown that the discriminative and representative ability of the stacked RBM for each type varies according to the provided amino acid property index combinations, but keeping, in general, a stable and consistent classification pattern across all index combinations. Moreover, and importantly for the problem of recognizing the subfamilies related to the odor function, the experimental results indicate that RBM in conjunction with any amino acid physicochemical property index combinations can quite accurately represent and discriminate the vomeronasal type, and specifically using the 485-247-193 index combination, it can also highly recognize the pheromone and odorant subfamilies.

Motivated by the fact that relevant features of two class C GPCR subfamilies (related to the odor function) are difficult to represent and classifiers confuse them, a multi-label learning approach that allows an instance to belong to different classes is considered as future work. Furthermore, a pertinent evaluation of the three hydrophobicity-related index combinations found in this work should be carried out at a biochemical level.

## Figures and Tables

**Table 1:** Distribution of the unaligned class C GPCRs.

Type	Number of seq.
*Calcium sensing*	46
$GABA_{B}$	193
*Metabotropic glutamate*	321
*Odorant*	91
*Pheromone*	372
*Taste*	65
*Vomeronasal*	304
Total	1392

**Table 2:** Distribution of the aligned class C GPCRs.

Type	Number of seq.
*Calcium sensing*	36
$GABA_{B}$	139
*Metabotropic glutamate*	296
*Odorant*	82
*Pheromone*	356
*Taste*	60
*Vomeronasal*	230
Total	1199

**Table 3:** Average classification accuracy (%) results of four classifiers using alignment-free representation datasets. AAcomp, amino acid composition; PseAA, pseudo-amino acid composition.

Transformation	MLP	SVM	DT	*k*-NN
AAcomp	83.98	87.64	72.13	84.99
ACC	89.08	**91.67**	61.35	87.79
PseAA	88.22	88.86	74.21	87.43
PseAA-MSE	87.57	88.51	72.49	88.00

**Table 4:** Average classification results using the amino acid hydrophobicity-related index (AAhydro) set.

Deep architecture	Accuracy (%)
Autoencoder	71.98
Convolutional neural network	71.68
Restricted Boltzmann machine	86.68

**Table 5:** Average classification results for an RBM with a hidden layer using the AAhydro set.

${\mathrm{layer}}_{1}$ (#Neurons)	Accuracy (%)
100	88.11
200	88.48
300	88.45
500	**89.33**
800	87.98

**Table 6:** Average classification accuracy results for an RBM with two hidden layers using the AAhydro set.

${\mathrm{Layer}}_{1}$	${\mathrm{Layer}}_{2}$				
	100	200	300	500	800
100	90.62	88.24	88.61	87.90	88.45
200	88.24	88.24	88.03	87.98	88.28
300	88.61	88.03	88.19	87.82	88.03
500	87.90	87.98	87.82	**89.71**	87.98
800	88.45	88.28	88.03	87.98	87.82

**Table 7:** Average classification accuracy results for an RBM with three hidden layers using the AAhydro set.

${\mathrm{Layer}}_{1},{\mathrm{Layer}}_{2}$	${\mathrm{Layer}}_{3}$		
	300	500	800
300, 300	79.06	77.23	78.32
300, 500	77.96	77.27	78.32
500, 300	76.50	78.69	77.23
500, 500	77.96	81.24	77.96
300, 800	83.07	83.07	81.24
800, 300	**83.43**	82.70	83.06
800, 800	78.69	79.42	79.42
500, 800	81.97	82.70	81.97
800, 500	80.15	78.32	83.07

**Table 8:** Average classification results for an RBM with four hidden layers using the AAhydro set.

${\mathrm{Layer}}_{1},{\mathrm{Layer}}_{2},{\mathrm{Layer}}_{3},{\mathrm{Layer}}_{4}$	Accuracy (%)
300, 300, 300, 300	**82.70**
500, 500, 500, 500	82.34
800, 800, 800, 800	81.24

**Table 9:** Average classification accuracy results for an RBM with two hidden layers using a fine grid search around 500 neurons and the AAhydro set.

${\mathrm{Layer}}_{1}$	${\mathrm{Layer}}_{2}$				
	400	450	500	550	600
400	81.24	81.97	81.61	81,60	81,60
450	84.16	77.96	79.41	81.60	80.51
500	83.80	80.51	**89.71**	81.60	79.41
550	79.78	79.05	79.78	81.24	82.70
600	82.70	79.78	79.05	79.05	81.24

**Table 10:** Highest average accuracy results of the restricted Boltzmann machine (RBM) with two hidden layers over 531 amino acid property indices. MCC, Matthews’ correlation coefficient; BER, balanced error rate.

Name	Index	Accuracy (%)	MCC	BER
Hydrophobicity index	531	92.86	91.12	7.71
Principal eigenvector of contact matrices and hydrophobicity profiles	485	92.82	91.02	10.33
Frequency of occurrence in beta-bends	166	92.40	90.41	10.69
Distinct character in hydrophobicity of the amino acid composition	193	91.93	89.83	9.88
Weights for coil at the window position of −2	288	91.81	89.68	11.78
NMR chemical shift of the alpha-carbon	84	91.76	89.68	13.34
AA composition of EXT2of single-spanning proteins	205	91.72	89.64	11.69
Relative mutability	65	91.60	89.50	10.52
Protein surface amino acid compositions	471	91.51	89.39	11.39
Hydrophobic packing and spatial arrangement of amino acids	247	90.97	88.90	9.89
Proportion of residues 95% buried	35	90.42	88.26	9.70
Normalized van der Waals volume	80	90.29	87.96	10.60

**Table 11:** Highest accuracy results of RBM using the amino acid property two-index combinations from Table 10.

Index	Accuracy (%)	MCC	BER
65-205	93.91	92.34	8.28
35-65	93.53	91.88	9.12
84-193	93.45	91.77	6.96
247-166	93.11	91.32	9.52
84-205	93.03	91.28	11.55

**Table 12:** Highest accuracy results of RBM using the amino acid property three-index combinations from Table 10.

Index	Accuracy (%)	MCC	BER
485-247-193	94.08	92.67	5.18
247-80-166	92.82	91.11	9.48
485-65-166	92.73	90.94	11.16
247-166-471	92.69	90.86	8.18
35-80-471	91.64	89.73	7.87

**Table 13:** Comparison of RBM results with conventional classification methods using one, two and three amino acid property index combinations.

Index	SVM			DT			*k*-NN			RBM		
Combination	Accu	MCC	BER	Accu	MCC	BER	Accu	MCC	BER	Accu	MCC	BER
531	90.99	87.80	11.77	87.16	82.07	15.79	89.41	86.25	11.43	**92.86**	91.12	7.71
65-205	91.24	88.04	11.73	88.57	83.78	13.65	90.49	87.71	10.30	**93.91**	92.34	8.28
485-247-193	90.74	87.34	12.96	88.32	83.78	12.97	90.33	87.65	10.33	**94.08**	92.67	5.18

## Supplementary Materials

The following are available online, Files S1–S26: datasets used in the experiments and described in the main text.

## References

[1] Katritch V., Cherezov V., Stevens R.C. (2013). Structure-Function of the G Protein–Coupled Receptor Superfamily. Annu. Rev. Pharmacol. Toxicol..

[2] DeVree B.T., Mahoney J.P., Vélez-Ruiz G.A., Rasmussen S.G.F., Kuszak A.J., Edwald E., Fung J.J., Manglik A., Masureel M., Du Y. (2016). Allosteric coupling from G protein to the agonist-binding pocket in GPCRs. Nature.

[3] Cahill T.J., Thomsen A.R.B., Tarrasch J.T., Plouffe B., Nguyen A.H., Yang F., Huang L.Y., Kahsai A.W., Bassoni D.L., Gavino B.J. (2017). Distinct conformations of GPCR—*β*-arrestin complexes mediate desensitization, signaling, and endocytosis. Proc. Natl. Acad. Sci. USA.

[4] Fredriksson R., Lagerström M.C., Lundin L.G., Schiöth H.B. (2003). The G-Protein-Coupled Receptors in the Human Genome Form Five Main Families. Phylogenetic Analysis, Paralogon Groups, and Fingerprints. Mol. Pharmacol..

[5] Cooke R.M., Brown A.J., Marshall F.H., Mason J.S. (2015). Structures of G protein-coupled receptors reveal new opportunities for drug discovery. Drug Discov. Today.

[6] Eddy M.T., Lee M.Y., Gao Z.G., White K.L., Didenko T., Horst R., Audet M., Stanczak P., McClary K.M., Han G.W. (2018). Allosteric Coupling of Drug Binding and Intracellular Signaling in the A2A Adenosine Receptor. Cell.

[7] Hill S.J., Watson S.P. (2018). Fluorescence Approaches Unravel Spatial and Temporal Aspects of GPCR Organisation, Location, and Intracellular Signalling. Trends Pharmacol. Sci..

[8] Hertig S., Latorraca N.R., Dror R.O. (2016). Revealing Atomic-Level Mechanisms of Protein Allostery with Molecular Dynamics Simulations. PLoS Comput. Biol..

[9] Sriram K., Insel P.A. (2018). GPCRs as targets for approved drugs: How many targets and how many drugs?. Mol. Pharmacol..

[10] Peng Y., McCorvy J.G., Harpsøe K., Lansu K., Yuan S., Popov P., Qu L., Pu M., Che T., Nikolajsen L.F. (2018). 5-*HT*_2*C*_ Receptor Structures Reveal the Structural Basis of GPCR Polypharmacology. Cell.

[11] Cruz-Barbosa R., Vellido A., Giraldo J. (2015). The influence of alignment-free sequence representations on the semi-supervised classification of class C G protein-coupled receptors. Med. Biol. Eng. Comput..

[12] König C., Cruz-Barbosa R., Alquézar R., Vellido A. (2013). SVM-Based Classification of Class C GPCRs from Alignment-Free Physicochemical Transformations of Their Sequences. Proceedings of the 17th New Trends in Image Analysis and Processing.

[13] Karchin R., Karplus K., Haussler D. (2002). Classifying G-protein coupled receptors with support vector machines. Bioinformatics.

[14] Rehman Z.U., Khan A. (2011). G-protein-coupled receptor prediction using pseudo-amino-acid composition and multiscale energy representation of different physiochemical properties. Anal. Biochem..

[15] Otaki J.M., Mori A., Itoh Y., Nakayama T., Yamamoto H. (2006). Alignment-Free Classification of G-Protein-Coupled Receptors Using Self-Organizing Maps. J. Chem. Inf. Model..

[16] Qiu J.D., Huang J.H., Liang R.P., Lu X.Q. (2009). Prediction of G-protein-coupled receptor classes based on the concept of Chou’s pseudo amino acid composition: An approach from discrete wavelet transform. Anal. Biochem..

[17] Liao Z., Ju Y., Zou Q. (2016). Prediction of G Protein-Coupled Receptors with SVM-Prot Features and Random Forest. Scientifica.

[18] Yang Y., Lu B., Yang W. Classification of protein sequences based on word segmentation methods. Proceedings of the 6th AsiaPacific Bioinformatics Conference.

[19] Liu B., Wang X., Chen Q., Dong Q., Lan X. (2012). Using Amino Acid Physicochemical Distance Transformation for Fast Protein Remote Homology Detection. PLoS ONE.

[20] König C., Alquézar R., Vellido A., Giraldo J. (2014). Reducing the n-gram feature space of class C GPCRs to subtype-discriminating patterns. J. Integr. Bioinform..

[21] Bengio Y., Courville A., Vincent P. (2013). Representation Learning: A Review and New Perspectives. IEEE Trans. Pattern Anal. Mach. Intell..

[22] Lin Z., Lanchantin J., Qi Y. MUST-CNN: A Multilayer Shift-and-Stitch Deep Convolutional Architecture for Sequence-based Protein Structure Prediction. Proceedings of the Thirtieth AAAI Conference on Artificial Intelligence (AAAI-16).

[23] Wei L., Ding Y., Su R., Tang J., Zou Q. (2017). Prediction of human protein subcellular localization using deep learning. J. Parallel Distrib. Comput..

[24] Mohamed A., Dahl G.E., Hinton G. (2012). Acoustic Modeling Using Deep Belief Networks. IEEE Trans. Audio Speech Lang. Process..

[25] Cadieu C.F., Hong H., Yamins D.L.K., Pinto N., Ardila D., Solomon E.A., Majaj N.J., DiCarlo J.J. (2014). Deep Neural Networks Rival the Representation of Primate IT Cortex for Core Visual Object Recognition. PLoS Comput. Biol..

[26] Cireşan D., Meier U., Masci J., Schmidhuber J. (2012). Multi-column deep neural network for traffic sign classification. Neural Netw..

[27] Kawashima S., Kanehisa M. (2008). AAindex: Amino acid index database, progress report 2008. Nucleic Acids Res..

[28] Pin J.P., Galvez T., Prézeau L. (2003). Evolution, Structure, and Activation Mechanism of Family 3/C G-protein-coupled receptors. Pharmacol. Ther..

[29] Kniazeff J., Prézeau L., Rondard P., Pin J.P., Goudet C. (2011). Dimers and beyond: The functional puzzles of class C GPCRs. Pharmacol. Ther..

[30] Isberg V., Vroling B., van der Kant R., Li K., Vriend G., Gloriam D. (2014). GPCRDB: An information system for G protein-coupled receptors. Nucleic Acids Res..

[31] Vroling B., Sanders M., Baakman C., Borrmann A., Verhoeven S., Klomp J., Oliveira L., de Vlieg J., Vriend G. (2011). GPCRDB: Information system for G protein-coupled receptors. Nucleic Acids Res..

[32] Wu H., Wang C., Gregory K.J., Han G.W., Cho H.P., Xia Y., Niswender C.M., Katritch V., Meiler J., Cherezov V., Conn P.J., Stevens R.C. (2014). Structure of a class C GPCR Metabotropic Glutamate Receptor 1 bound to an allosteric modulator. Science.

[33] Doré A.S., Okrasa K., Patel J.C., Serrano-Vega M., Bennett K., Cooke R.M., Errey J.C., Jazayeri A., Khan S., Tehan B. (2014). Structure of class C GPCR metabotropic glutamate receptor 5 transmembrane domain. Nature.

[34] Edgar R.C. (2004). MUSCLE: Multiple sequence alignment with high accuracy and high throughput. Nucleic Acids Res..

[35] Sievers F., Wilm A., Dineen D., Gibson T.J., Karplus K., Li W., Lopez R., McWilliam H., Remmert M., Söding J. (2011). Fast, scalable generation of high-quality protein multiple sequence alignments using Clustal Omega. Mol. Syst. Biol..

[36] Notredame C., Higgins D.G., Heringa J. (2000). T-coffee: A novel method for fast and accurate multiple sequence alignment. J. Mol. Biol..

[37] Lapinsh M., Gutcaits A., Prusis P., Post C., Lundstedt T., Wikberg J.E. (2002). Classification of G-protein coupled receptors by alignment-independent extraction of principal chemical properties of primary amino acid sequences. Protein Sci..

[38] Wold S., Jonsson J., Sjörström M., Sandberg M., Rännar S. (1993). DNA and peptide sequences and chemical processes multivariately modelled by principal component analysis and partial least-squares projections to latent structures. Anal. Chim. Acta.

[39] Chou K.C. (2001). Prediction of protein cellular attributes using pseudo-amino acid composition. Proteins.

[40] Chou K.C. (2005). Using amphiphilic pseudo amino acid composition to predict enzyme subfamily classes. Bioinformatics.

[41] Chou K.C., Cai Y.D. (2005). Prediction of Membrane Protein Types by Incorporating Amphipathic Effects. J. Chem. Inf. Model..

[42] Shen H.B., Chou K.C. (2008). PseAAC: A flexible web server for generating various kinds of protein pseudo amino acid composition. Anal. Biochem..

[43] Fauchereand J., Pliska V. (1983). Hydrophobic parameters of amino-acid side chains from the partitioning of *N*-acetyl-amino-acid amides. Eur. J. Med. Chem..

[44] Sandberg M., Eriksson L., Jonsson J., Sjöström M., Wold S. (1998). New Chemical Descriptors Relevant for the Design of Biologically Active Peptides. A Multivariate Characterization of 87 Amino Acids. J. Med. Chem..

[45] LeCun Y., Bengio Y., Hinton G. (2015). Deep learning. Nature.

[46] Bengio Y. (2009). Learning Deep Architectures for AI. Found. Trends Mach. Learn..

[47] Vincent P., Larochelle H., Bengio Y., Manzagol P., Cohen W., McCallum A., Roweis S. (2008). Extracting and composing robust features with denoising autoencoders. Proceedings of the Twenty-fifth International Conference on Machine Learning (ICML’08).

[48] Hinton G.E., Osindero S., Teh Y.W. (2006). A Fast Learning Algorithm for Deep Belief Nets. Neural Comput..

[49] Vapnik V.N. (1998). Statistical Learning Theory.

[50] Ben-Hur A., Ong C.S., Sonnenburg S., Schölkopf B., Rätsch G. (2008). Support Vector Machines and Kernels for Computational Biology. PLoS Comput. Biol..

[51] Gorodkin J. (2004). Comparing two K-category assignments by a K-category correlation coefficient. Comput. Biol. Chem..

[52] Jurman G., Riccadonna S., Furlanello C. (2012). A comparison of MCC and CEN Error Measures in Multi-Class Prediction. PLoS ONE.

[53] Witten I.H., Frank E., Hall M.A. (2011). Data Mining: Practical Machine Learning Tools and Techniques.

[54] Rong X. Deepnet: Deep Learning Toolkit in R. https://cran.r-project.org/web/packages/deepnet/index.html.

[55] Software-Foundation A. MXNet-R API. https://mxnet.incubator.apache.org/api/r/index.html.

[56] Sutskever I. (2013). Training Recurrent Neural Networks. Ph.D. Thesis.

